# Photoacoustic Spectral Analysis for Evaluating the Aggressiveness of Prostate Cancer Labeled by Methylene Blue Polyacrylamide Nanoparticles

**DOI:** 10.3390/bios13030403

**Published:** 2023-03-20

**Authors:** Janggun Jo, Eamon Salfi, Jeff Folz, Aaron M. Udager, Evan Keller, Raoul Kopelman, Sri-Rajasekhar Kothapalli, Guan Xu, Xueding Wang

**Affiliations:** 1 Department of Biomedical Engineering, University of Michigan, Ann Arbor, MI 48109, USA; 2 Department of Chemistry, University of Michigan, Ann Arbor, MI 48109, USA; 3 Department of Pathology, University of Michigan Health System, Ann Arbor, MI 48109, USA; 4 Department of Urology, University of Michigan, Ann Arbor, MI 48109, USA; 5 Department of Biomedical Engineering, Pennsylvania State University, University Park, PA 16801, USA; 6 Department of Ophthalmology and Visual Sciences, University of Michigan, Ann Arbor, MI 48109, USA

**Keywords:** photoacoustic imaging, photoacoustic spectral analysis, prostate cancer, cancer aggressiveness, nanoparticle contrast agent, TRAMP model

## Abstract

Evaluating the aggressiveness of prostate cancer (PCa) is crucial for PCa diagnosis and prognosis. Previously, studies have shown that photoacoustic spectral analysis (PASA) can assess prostate tissue microarchitecture for evaluating the aggressiveness of PCa. In this study, in a transgenic mouse (TRAMP) model of PCa, we utilized methylene blue polyacrylamide nanoparticles (MB PAA NPs) to label the cancer cells in prostate in vivo. MB PAA NPs can specifically target proliferating cancer cells as a contrast agent, allowing photoacoustic (PA) imaging to better detect PCa tumors, and also assessing prostate glandular architecture. With the PA signals from the prostates measured simultaneously by a needle hydrophone and a PA and ultrasound (US) dual-imaging system, we conducted PASA and correlated the quantified spectral parameter slopes with the cancer grading from histopathology. The PASA results from 18 mice showed significant differences between normal and cancer, and also between low-score cancer and high-score cancer. This study in the clinically relevant TRAMP model of PCa demonstrated that PA imaging and PASA, powered by MB PAA NPs that can label the PCa microarchitectures in vivo after systemic administration, can detect PCa and, more importantly, evaluate cancer aggressiveness.

## 1. Introduction

Prostate cancer (PCa) is the most commonly diagnosed cancer in American men, with an annual incidence rate substantially higher than any other cancer [1,2]. PCa has a relatively high survival rate for patients with early diagnosis; however, the survival rate decreases dramatically once the cancer has metastasized [3,4]. Therefore, differentiating high-score from low-score PCa is important for preventing metastasis of PCa and improving patient outcome. The standard diagnostic procedure for PCa is transrectal ultrasound (TRUS)-guided biopsy [5], where small tissue pieces are extracted from the prostate. After histology processing, the microscopic architecture in the biopsy tissues is scored using a Gleason grading system as a reflection of the aggressiveness of PCa [6]. Due to the limited sensitivity of TRUS to PCa, less than 10% of the sampled cores are clinically significant [7,8], and the core extractions are frequently accompanied by post-procedure complications [9].

With the unique capability to present highly sensitive optical contrast in deep tissue with high temporal and spatial resolution, non-ionizing and non-invasive photoacoustic (PA) imaging has drawn considerable attention during the past two decades. When integrated with conventional ultrasound (US) imaging, PA imaging based on endogenous contrast among tissues or powered by optical contrast agents is capable of providing additional functional and molecular information which are both highly valuable for understanding the onset, progress, and responses to therapy of diseases such as cancer [10,11,12,13,14,15]. PA imaging at ultraviolet (UV) wavelengths has shown the capability of producing histology-like images of the microscopic architectures in tissues [16]. Our recent study has shown that, by quantitatively analyzing the power distribution in the frequency domain of the PA signals acquired at UV wavelengths, i.e., PA spectral analysis (PASA), the Gleason grades of ex vivo prostate tissues can be assessed [17]. However, the limited penetration of UV light narrows its potential for non-invasive applications. On the other hand, simultaneous US and PA imaging at near-infrared (NIR) wavelengths has succeeded in penetrating the rectum wall and identifying malignant regions in human prostates in vivo [18], although the imaging procedure did not involve the assessment of prostate microarchitecture for evaluating the aggressiveness of malignancy. We expect that a combination of PA-US imaging with PASA may enable not only PCa detection, but also an assessment of PCa aggressiveness in vivo.

Our previous studies have also shown that F3-peptide-conjugated polyacrylamide nanoparticles (PAA NPs) can target the nucleolin in proliferating cancer cells [14,15,19,20]. In this study, we explored the use of F3-conjugated PAA NPs loaded with methylene blue (MB) dye to label the PCa cells in vivo. With the PCa glandular architecture labeled by the PAA NPs as the contrast agent, PA imaging of PCa tumors and a PASA-based assessment of PCa aggressiveness became possible. To validate the feasibility of this method, experiments were conducted in a clinically relevant transgenic mouse (TRAMP) model of PCa.

## 2. Materials and Methods

### 2.1. Preparation of MB-PAA NPs

Unless otherwise noted, all materials were purchased from Sigma-Aldrich (St. Louis, MO, USA). All chemicals were used without further purification. MB-PAA NPs were prepared using a water-in-oil emulsion and free radical polymerization. Descriptions of this method can be found in our previously published work [21].

A monomer solution was prepared, containing 368 mg of acrylamide and 28 mg of N-(3aminopropyl)-methacrylamide dissolved in 0.93 mL of phosphate-buffered saline solution (PBS). Then, 5 mg dicarboxymethylene blue NHS ester (DCMB-SE), dissolved in 100 µL dimethyl sulfoxide (DMSO), was added to the monomer solution and stirred for 2 h. An oil phase was then prepared consisting of 1.07 g of sodium dioctyl sulfosuccinate, 2.2 mL of Brij L4, and 30 mL of hexanes. This solution was then stirred and purged with argon gas for 30 min. Next, 200 µL of 3-(acryloyl)-2-hydroxypropyl methacrylate was added to the monomer solution, before it was mixed by vortexing and injected into the oil phase. The oil phase must be further purged with argon for at least 20 additional minutes before radical polymerization can be initiated, and must be conducted under an inert atmosphere. Polymerization was initiated, firstly by injecting 100 µL of N,N,N′,N′-tetramethylethylenediamine into the reaction vessel, followed by 100 µL of a 10% (*w*/*v*) solution of ammonium persulfate in PBS. The reaction was then stirred for 2 h under argon.

Following the reaction, the hexanes were removed using rotary evaporation. MB-PAA NPs were then resuspended in ethanol and transferred to an Amicon stirred ultrafiltration cell equipped with a Biomax 300 kDa membrane filter. The MB-PAA NP solution was washed 5 times with ethanol, followed by 5 additional washes with Millipore water. Next, the MB-PAA NPs were lyophilized and stored at −20 °C. To actively target MB-PAA NPs to the tumor area, the nanoparticles’ surface was conjugated with F3 peptides. A PBS solution (pH 7.4, 2.5 mL) containing MB-PAA NPs (50 mg) and bifunctional polyethylene glycol (MAL-PEG-SCM, 2 kDa, Creative PEGWorks) (4 mg) was stirred for 30 min. The mixture was then washed 3 times with PBS using an Amicon ultra-centrifugal filter (100 kDa) to remove unreacted PEG. After the washes, the solution was resuspended to its original concentration (50 mg/2.5 mL) and F3 peptides (KDEPQRRSARLSAKPAPPKPEPKPKKAPAKKC, RS Synthesis) (11 mg) were added. The reaction was stirred overnight. Cysteine (0.63 mg) was added to quench the unreacted maleimide groups, and was then stirred for 2 h. The solution was then washed again to remove unreacted F3 and cysteine, before being lyophilized.

**Figure 1:**
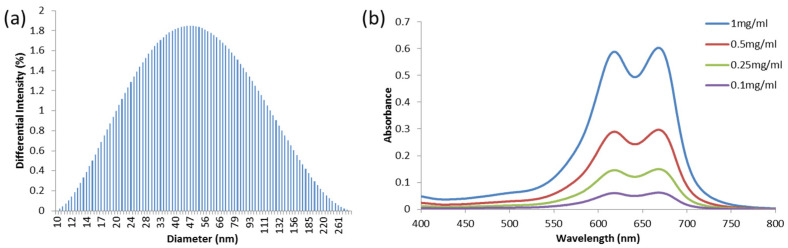
(**a**) Dynamic light scattering (DLS) of MB PAA NPs. (**b**) UV-vis absorption spectra of MB PAA NPs in PBS pH 7.4 buffer for different concentrations (0.1, 0.25, 0.5, and 1 mg/mL).

**Figure 2:**
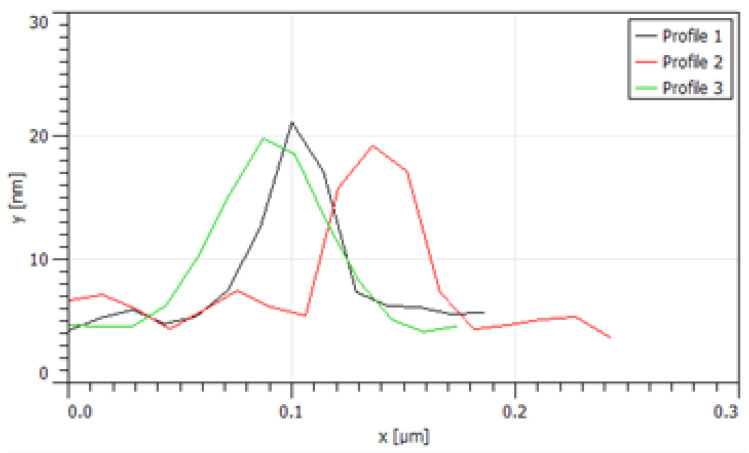
Three traces of the nanoparticle diameters collected via atomic force microscopy. The NPs are shown to be around 60 nm in diameter.

Dynamic light scattering (DynaPro Nanostar, Wyatt Technology, Santa Barbara, CA, USA) was used to determine the particle size. A spectrometer (UV-1800, Shimadzu, Kyoto, Japan) was used for absorption spectra and a FluoroMax-3 spectrofluorometer (Jobin Yvon Horiba, Kyoto, Japan) was used for fluorescence spectra. Both spectrometers were used to determine the dye loadings and NP characteristics, compared to the previously reported MB-PAA NPs [21]. The average size distribution was 73.7 nm (±6.8 nm), as shown in Figure 1a, which matches the previously reported hydrodynamic size, 78.5 nm (±5.8 nm) [21]. The optical absorption spectra at different concentrations of MB PAA NPs in PBS pH 7.4 buffer are shown in Figure 1b. The atomic force microscopy traces (Figure 2) are in good agreement with the DLS characterization. The AFM measurements show nanoparticles with diameters near 60 nm.

### 2.2. Animal Model

In the study, we used a transgenic mouse strain, TRAMP (008215, Jackson Laboratory, Bar Harbor, ME, USA), that spontaneously develops PCa with microscopic architecture close to that in human PCa [22]. The laboratory animal protocol for this study was approved by the Institutional Animal Care and Use Committee (IACUC) at the University of Michigan (Animal protocol: PRO00009561). In total, 18 TRAMP mice were used in the study. To obtain PCa tumors at different stages, the TRAMP mice involved in this study had ages ranging from 6 to 18 weeks when utilized in contrast agent injection and PA imaging and measurement. Our goal was to perform PA imaging and measure the prostates at three different conditions: noncancerous control, early-stage cancer, and late-stage cancer. Since the cancer progression of TRAMP mice is not uniform compared to their age, the condition of each prostate was revealed by histopathology procedure as the gold standard, and then compared with the result from PASA.

### 2.3. Experimental Procedure

Each TRAMP mouse was anesthetized with the inhalation of 1.0–2.0% isoflurane mixing with oxygen. The MB PAA NP solution (20 mg/mL in saline) was injected through the tail vein, with a dose of 250 mg NPs per kg body weight. The mouse was euthanized at 60 min after the MB PAA NP injection. According to our previous study [15], the PAA NPs conjugated with F3 peptides have the highest accumulation in the prostate at this time point following injection. The prostate of each animal was harvested for ex vivo imaging and measurement.

**Figure 3:**
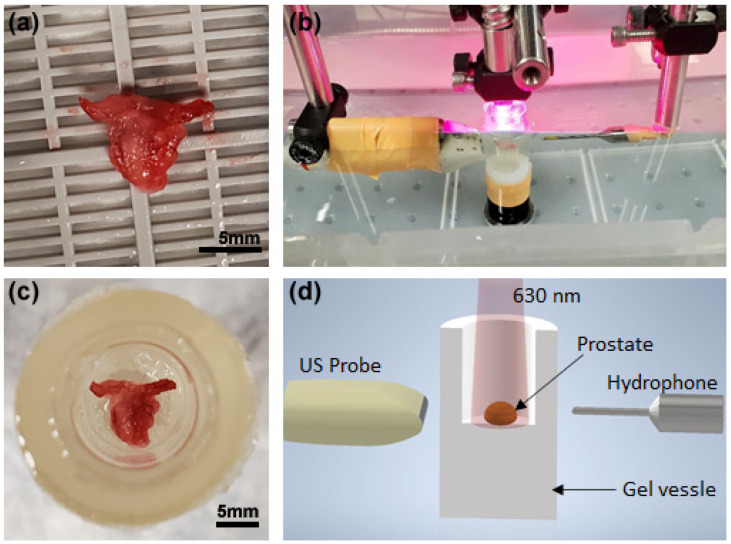
Experimental setup for PA measurement and PA-US dual imaging of TRAMP mouse prostate ex vivo. (**a**) A harvested prostate from a TRAMP mouse at the age of 18 weeks. (**b**) Photograph of experimental setup. (**c**) Prostate embedded in a porcine vessel for US coupling. (**d**) Experimental setup schematic drawing, showing that the PA signals in the prostate induced by the 630 nm laser are measured by a needle hydrophone as well as by a linear US probe for PA-US imaging.

Figure 3 shows the experimental setup. The harvested prostate was put into a gel vessel made from porcine skin powder and placed in water for acoustic coupling, as shown in Figure 3c,d. An optical parameters oscillator (SLOPO, Continuum, Santa Clara, CA, USA), pumped by an Nd:YAG laser (Surelite, Continuum, Santa Clara, CA, USA), was used as the illumination source for PA imaging and measurement. The laser wavelength was tuned to 630 nm. At this wavelength, the MB dye has strong optical absorption, as shown in Figure 1b, while the optical absorption from the intrinsic chromophores in tissues, especially hemoglobin, is relatively weak. The laser-induced PA signals were captured by a needle hydrophone with frequency response calibrated within 0.1–20 MHz (HNC-1500 and preamplifier AH2010-025, ONDA corporation, Sunnyvale, CA, USA) at a sampling rate of 250 MHz. At the same time as PA measurement using the hydrophone, PA signals from the prostate were also detected by a PA and US dual-modality imaging system built on a commercially available research US platform (V1, Verasonics, Kirkland, WA, USA), as shown in Figure 3b,d. This system allows PA and US imaging of a biology sample simultaneously, both in real time. The PA and US images of the prostate were acquired using a linear probe (L22-14, Verasonics, Kirkland, WA, USA) working at the default central frequency of 18 MHz. In this study, each prostate was detected along one position only by the needle hydrophone and the US probe. Due to the small volume of mouse prostate, the PA signals measured here for PASA were from the entire prostate.

### 2.4. Photoacoustic Spectral Analysis (PASA)

With the PA signals acquired by the calibrated needle hydrophone, the power spectra of the signals were analyzed in the frequency domain, following the method described in our previous publication [17]. Briefly, a linear fit of each power spectrum within the range of 5–20 MHz was performed, and the spectral parameter *slope* was calculated. To improve the signal-to-noise ratio, the average over 16 PA waveforms from the hydrophone was used to calculate the spectral parameter *slope*. Besides working on the PA signals acquired by the needle hydrophone, PASA was performed on the PA signals acquired by the PA-US dual imaging system. The L22-14 probe has a total of 128 transducer elements. The radio frequency PA signals acquired by the central 50 transducer elements, which were directly facing the prostate during the imaging procedure, were analyzed. The power spectrum of the PA signal from each transducer element was generated, and then the power spectra from all 50 central transducer elements were averaged to improve the signal-to-noise ratio. Then, a linear fit of the averaged power spectrum was performed within the range of 5–20 MHz, which led to the quantified spectral parameter *slope*.

### 2.5. Histology

After imaging and measurement, each prostate sample was processed for standard histology. The prostate was embedded in paraffin and sliced at a step of 200 µm. At each step, two 26 µm sections were sliced. One of the slices was stained with hematoxylin and eosin (H&E). The other was not stained, in order to allow for later fluorescence microscopy imaging. Under the guidance of board-certified pathologists, each prostate was assigned with a grade in the range of 0–7, following the method described in the literature [23]. Grade 0 is considered normal before cancer development, Grades 1–2 indicate low-score PCa, while Grades 3–7 indicate high-score PCa.

## 3. Results

### 3.1. PCa Microarchitectures Labeled by MB PAA NPs

**Figure 4:**
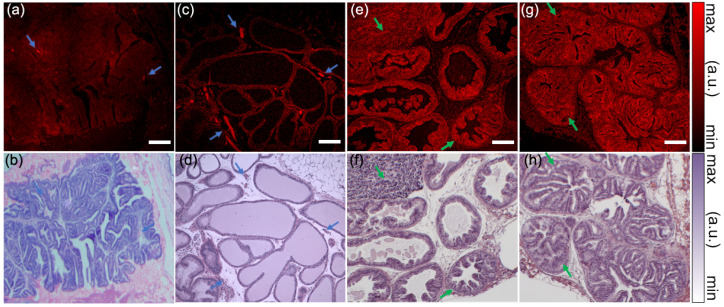
Microarchitectures of PCa in TRAMP mice labeled in vivo by F3-conjugated PAA NPs loaded with fluorescent MB dye. The top row shows the fluorescence images, and the bottom row shows the paired H&E stained histology images. (**a**,**b**) Cancerous prostate without NP injections. (**c**,**d**) Normal prostate with NP injection. (**e**–**h**) Cancerous prostates, both with NP injections. The blue arrows mark the red blood cells with auto-fluorescence. The green arrows correspond to the glandular microarchitectures at progressive cancer stages. Images are taken at 10× magnification. Scale bar: 100 µm.

Figure 4a shows a cancerous prostate tissue, as confirmed by Figure 4b, without MB-PAA NP injection. Without NP staining, the autofluorescence emitted by the cells in the cancerous prostate has magnitudes lower than that emitted by the red blood cells. Figure 4c shows a normal prostate tissue, as confirmed by Figure 4d, with MB-PAA NP injection. Since the MB-PAA NPs do not target the normal cells, the autofluorescence emitted by the normal prostate cells also has magnitudes lower than that emitted by red blood cells. Figure 4e,g show the appearance of the MB-PAA NPs in the cancerous prostate tissues. Figure 4f,h show the matching histology with the H&E stain, showing the glandular architectures at progressive cancer stages. In contrast with the control imaging in Figure 4a,c, the cancer cells in Figure 4e,g, due to the targeted labeling by MB-PAA NPs, show overwhelming fluorescence contrast compared to other tissue components.

### 3.2. PA-US Dual Imaging of Prostate

**Figure 5:**
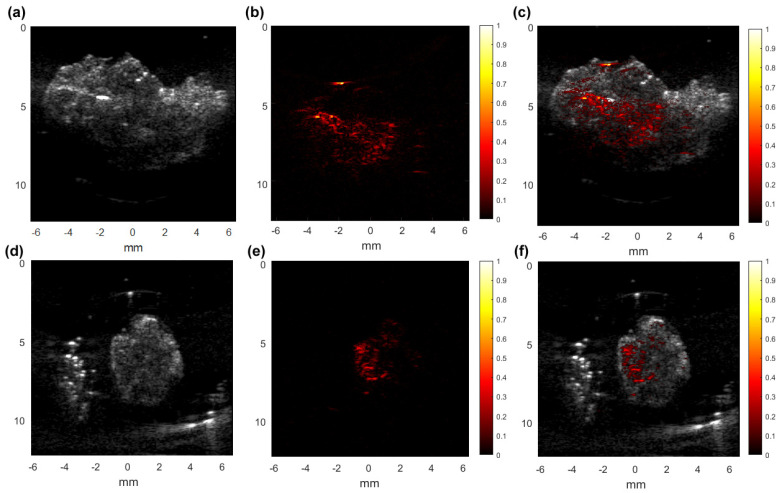
PA and US images of a cancerous mouse prostate (top line) vs. a normal mouse prostate (bottom line). (**a**) Gray–scale US image of the cancerous prostate. (**b**) Pseudo–color PA image at 630 nm obtained from the same imaging plane of (**a**). The dominant signals are from the F3–conjugated MB PAA NPs that specifically target cancer cells. (**c**) The PA image in (**b**) superimposed on the US image in (**a**). (**d**) Gray–scale US image of the normal prostate. (**e**) Pseudo–color PA image at 630 nm obtained from the same imaging plane of (**d**). (**f**) The PA image in (**e**) superimposed on the US image in (**d**).

Figure 5 shows example PA-US dual imaging results from a cancerous prostate harvested from a TRAMP mouse, and a normal mouse prostate. The gray-scale US images show the macroscopic tissue structures. Because the dominant PA signals at the 630 nm wavelength are from the MB-PAA NPs, the pseudo-color PA images at this wavelength confirm the spatially distributed MB-PAA NPs in the prostates. Comparing the PA signals in the normal prostate (Figure 5e,f), the PA signals in the cancerous prostate (Figure 5b,c) are stronger when the same color bar is used for the PA images.

### 3.3. PASA of the Signals from Prostate

#### 3.3.1. PASA of the Results from the Needle Hydrophone

**Figure 6:**
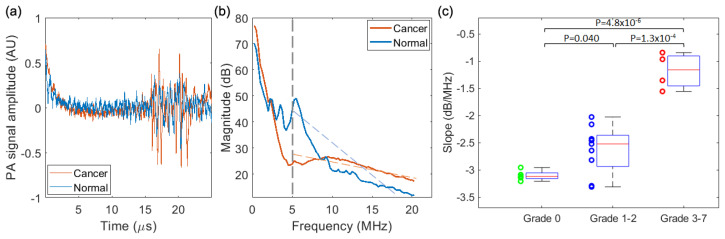
PASA results from the prostates of 18 TRAMP mice acquired by the needle hydrophone. (**a**) PA signals from a cancerous prostate and a normal prostate. (**b**) Power spectra of the PA signals from the cancerous prostate and the normal prostate. The linear fit of the power spectrum in the range of 5–20 MHz leads to the spectral parameter *slope*. (**c**) Quantified spectral parameter *slopes* of the prostates categorized in three groups, including Grade 0, Grades 1–2, and Grades 3–7, based on pathological evaluation. Two-tailed *t*-tests between the groups were performed, and those with statistically significant differences are marked in (**c**).

Figure 6 shows the PASA of results acquired by the needle hydrophone. The radio frequency PA signals and their corresponding power spectra from a cancerous prostate and a normal prostate, as two examples, are compared in Figure 6a,b. Compared to the normal prostate, the cancerous prostate shows stronger power in the high frequency range (>10 MHz). By performing a linear fit of the power spectrum in the range of 5–20 MHz, we quantified the spectral parameter *slope* of each of the 18 prostates, as Figure 6c. The 18 prostates are categorized into three groups according to their grades based on pathological evaluation, including Grade 0 for normal prostates, Grades 1–2 for low-score PCa, and Grades 3–7 for high-score PCa. The averages and the standard deviation of the *slopes* in the three groups are −−3.10 ± 0.09 (dB/MHz), −2.63 ± 0.44 (dB/MHz), and −1.18 ± 0.33 (dB/MHz), respectively. The *slopes* between any two of the three groups are compared by a two-tailed *t*-test performed using the built-in functions of MATLAB (R2021a, Mathworks, Natick, MA, USA). As shown in Figure 6c, there are statistically significant differences between Grade 0 and Grades 1–2 (*p* = 0.04), between Grades 1–2 and Grades 3–7 (*p* = 1.3 × 10^−4^), and between Grade 0 and Grades 3–7 (*p* = 4.8 × 10^−6^), demonstrating that the PASA of the PA signals acquired by the needle hydrophone is able to differentiate between the normal prostates and the cancerous prostates, and also between the low-score PCa and the high-score PCa.

#### 3.3.2. PASA of the Results from the PA-US Imaging System

**Figure 7:**
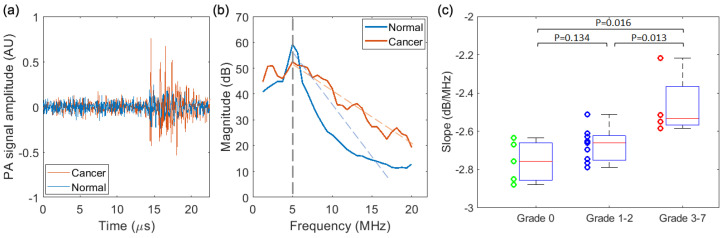
PASA results from the prostates of 18 TRAMP mice acquired by the PA–US dual imaging system. (**a**) PA signals from a cancerous prostate and a normal prostate. (**b**) Power spectra of the PA signals from the cancerous prostate and the normal prostate. (**c**) Quantified spectral parameter *slopes* of the prostates categorized in three groups, including Grade 0, Grades 1–2, and Grades 3–7, based on pathological evaluation. Two–tailed *t*-tests between the groups were performed, and those with statistically significant differences are marked in (**c**).

Figure 7 shows the PASA of results acquired by the PA-US dual-modality imaging system. The radio frequency PA signals and their corresponding power spectra from a cancerous prostate and a normal prostate, as two examples, are compared in Figure 7a,b. As with the results from the needle hydrophone, the cancerous prostate shows stronger power in the high frequency range when compared to the normal prostate. For each prostate, the power spectra of the radio frequency signals from the central 50 transducer elements were averaged, and then the linear fit of the averaged power spectrum in the range of 5–20 MHz led to the quantified spectral parameter *slope* for each prostate. The *slopes* of all the 18 prostates are shown in Figure 7c, which are also categorized into three groups, including Grade 0, Grades 1–2, and Grades 3–7. The averages and the standard deviation of the *slopes* in the three groups are −−2.76 ± 0.11 (dB/MHz), −2.67 ± 0.09 (dB/MHz), and −2.46 ± 0.17 (dB/MHz), respectively. The *slopes* between any two of the three groups are compared using the same two-tailed *t*-test. As shown in Figure 7c, there are statistically significant differences between Grades 1–2 and Grades 3–7 (*p* = 0.013), and between Grade 0 and Grades 3–7 (*p* = 0.016). There is also some difference between Grade 0 and Grades 1–2. However, the difference is not statistically significant (*p* = 0.134). Nevertheless, based on both the results from the needle hydrophone and the PA-US imaging system, PASA is able to distinguish between low-score PCa and high-score PCa.

## 4. Discussion and Conclusions

Using the clinically relevant TRAMP model of PCa, our results indicate a preferential accumulation of intravenously injected F3-conjugated MB PAA NPs in cancerous prostates, compared to normal prostates. Because F3 peptides target general proliferating cells, MB PAA NPs conjugated with F3 can label the PCa cells and thereby enhance the PA contrast of the glandular structures in vivo. This is confirmed by fluorescence imaging, which shows that the contrast enhancement is limited to the cancer cells in the glandular structures, instead of the irrelevant connective tissues. With the contrast of the glandular structures enhanced by the MB PAA NPs, PA imaging of PCa, powered by the newly developed PASA technique, will have access to substantially enriched diagnostic information. As demonstrated by the results from this study, PASA is capable of differentiating between normal prostates and cancerous prostates, and, more importantly, between low-score and high-score PCa. This capability in assessing PCa aggressiveness could be highly valuable in clinical management of PCa, e.g., facilitating personalized treatment with improving patient outcomes.

Although the experiments in this study were conducted in a small -animal model of PCa, the presented PA imaging and PASA, powered by the cancer-targeting NP contrast agent, also holds potential for clinical translation. The PA imaging and measurement systems in this study showed sufficient frequency bandwidth for covering signal components representing the architectural information in the prostate at different cancer stages. Frequency-dependent acoustic attenuation in tissue plays a critical role in PASA. In this study, we fixed the US probe at a position of 1–2 cm away from each sample. This distance is comparable to that between the US transducer surface to the peripheral zone in the human prostate, where most cancer occurs, in a TRUS imaging scenario [18]. On the other hand, the PAA NPs used in this study are non-toxic, as demonstrated in our previous study [24], which facilitates their potential application in clinic. Using optically absorbing dyes encapsulated by the PAA NPs as the contrast agents offers more advantages than using free dyes. These PAA NPs can preserve the optical property of the dye molecules encapsulated into the nanoparticles, while protecting the dye from other biological micromolecules [15]. By PEGylating the surface of the MB PAA NPs and conjugating them with tumor-homing F3 peptides, the PAA NPs also demonstrate good levels of biocompatibility and are highly efficient at cancer targeting.

There are also some limitations with this study. One of them is that the mouse prostates were imaged and measured ex vivo instead of in vivo. The reason for this arrangement is to see the glandular structures in their early stages, as opposed to the large and solid tumors at late stages [25]. At early stages, the TRAMP mouse prostate is small and difficult to image without being harvested from the body. Another limitation of this study is the relatively small sample size. Due to this, we divided the samples into three groups, among which statistically significant differences were observed. When the sample size is larger and there are sufficient samples in each subdivided group, we expect that the PA imaging powered by PASA, as demonstrated in this study, could enable finer grading of PCa.

## Data Availability

The data that support the finding of this study are available from the corresponding author on request.
